# Artificial Intelligence and Esthetics: Redefining Precision and Beauty in Plastic Surgery

**DOI:** 10.3390/medicina62040633

**Published:** 2026-03-26

**Authors:** Dinu Iuliu Dumitrascu, Stefan Lucian Popa, Victor Incze, Darius-Stefan Amarie, Leo Gaspari, Paul Aluas, Abdulrahman Ismaiel, Daniel Corneliu Leucuta, Liliana David, Florin Vasile Mihaileanu, Claudia Diana Gherman, Vlad Dumitru Brata, Irina Dora Magurean

**Affiliations:** 1 Department of Anatomy, “Iuliu Hatieganu” University of Medicine and Pharmacy, 400000 Cluj-Napoca, Romania; d.dumitrascu@yahoo.com; 2 2nd Medical Department, “Iuliu Hatieganu” University of Medicine and Pharmacy, 400000 Cluj-Napoca, Romania; abdulrahman.ismaiel@yahoo.com (A.I.); lilidavid2007@yahoo.com (L.D.); 3 Faculty of Medicine, “Iuliu Hatieganu” University of Medicine and Pharmacy, 400000 Cluj-Napoca, Romania; vicincze@yahoo.com (V.I.); dariusamarie27@gmail.com (D.-S.A.); leogaspari@hotmail.fr (L.G.); paulaluas@hotmail.com (P.A.); 4 Department of Medical Informatics and Biostatistics, “Iuliu Hatieganu” University of Medicine and Pharmacy, 400349 Cluj-Napoca, Romania; dleucuta@umfcluj.ro; 5 Department of Surgery, Emergency County Hospital Cluj, “Iuliu Hatieganu” University of Medicine and Pharmacy, 400347 Cluj-Napoca, Romania; ms26rfl@yahoo.com; 6 Department of Surgery-Practical Abilities, “Iuliu Hatieganu” University of Medicine and Pharmacy, 400337 Cluj-Napoca, Romania; gherman.claudia@umfcluj.ro; 7 Department of Gastroenterology, Regional Institute of Gastroenterology and Hepatology “Prof. Dr. Octavian Fodor”, 400394 Cluj-Napoca, Romania; brata_vlad@yahoo.com; 8 Department Photo-Video, University of Arts and Design, 400148 Cluj-Napoca, Romania; irina.dora@gmail.com

**Keywords:** artificial intelligence, plastic surgery, preoperative planning, surgical outcomes

## Abstract

Artificial intelligence (AI) is increasingly reshaping esthetic and reconstructive plastic surgery by improving measurement accuracy, treatment planning, and prediction of surgical outcomes. This article provides a scientific overview of current AI applications, including automated image analysis, machine-learning-based outcome forecasting, and generative models for preoperative simulation. AI-driven three-dimensional morphometrics allow precise, reproducible quantification of facial and body structures, supporting more objective assessments of symmetry, proportion, and contour. Predictive algorithms trained on large clinical datasets can estimate postoperative results and complication risks with higher consistency than traditional subjective evaluation. Intraoperative AI tools, such as real-time image guidance and robotic assistance, show potential to increase procedural precision and reduce variability. Despite these advances, important limitations persist. Algorithmic bias, restricted data diversity, opaque model architectures, and unresolved ethical concerns regarding data privacy and esthetic standardization challenge widespread clinical adoption. Overall, AI offers a powerful framework for enhancing precision and reproducibility in esthetic surgery, but its safe and responsible integration will require rigorous validation, transparent methodology, and continued human oversight.

## 1. Introduction

Artificial intelligence (AI) has emerged as a transformative force across multiple medical disciplines, offering unprecedented capabilities in data analysis, predictive modeling, and decision support [1]. Within the field of plastic surgery, where precision, individualized planning, and esthetic outcomes are paramount, AI technologies are increasingly being explored to augment clinical judgment and optimize procedural results [2]. Recent advances in machine learning, computer vision, and neural network architectures have facilitated applications ranging from preoperative imaging analysis and outcome prediction to virtual surgical simulations and automated assessment of esthetic proportions [3].

Despite the growing integration of AI into clinical practice, its role in esthetic surgery remains incompletely characterized. The heterogeneity of available technologies, variability in methodological quality, and lack of standardized outcome measures pose significant challenges to clinicians seeking evidence-based guidance. A review of the literature is therefore essential to elucidate current applications, assess clinical efficacy, and identify limitations and future directions for AI-driven interventions in esthetic plastic surgery.

This review aims to evaluate existing evidence regarding AI applications in esthetic surgery, focusing on their impact on precision, reproducibility, and patient-centered outcomes. By critically analyzing published studies, this work seeks to provide a comprehensive framework for understanding how AI is redefining the intersection of technology and artistry in the pursuit of optimal esthetic results.

Esthetic plastic surgery demands an intricate balance between functional restoration and visual harmony. Traditionally, surgical planning has relied on surgeon experience, anthropometric measurements, and subjective assessment of patient-specific facial or bodily features. While these approaches have achieved high levels of artistry and technical success, they are inherently limited by inter-observer variability and the challenge of predicting postoperative outcomes with precision.

Artificial intelligence, encompassing machine learning algorithms, deep learning networks, and computer vision techniques, offers the potential to enhance decision-making in this domain. AI-driven tools can process large datasets of pre- and postoperative images, quantify anatomical landmarks with high accuracy, and generate predictive models for esthetic outcomes [4]. These technologies promise not only to standardize surgical planning and assessment but also to provide individualized recommendations that align with patient preferences and anatomical constraints.

Recent studies have demonstrated applications such as automated facial feature recognition, simulation of surgical modifications, and outcome prediction for procedures including rhinoplasty, breast augmentation, and facial rejuvenation. Despite these promising developments, the integration of AI into esthetic practice remains at an early stage, with variable evidence quality, limited validation, and ongoing ethical and regulatory considerations.

The primary objective of this narrative review is to critically evaluate the current literature on AI applications in esthetic plastic surgery, with a focus on:

- Identifying the types of AI technologies utilized and their specific clinical applications.
- Assessing the accuracy, reliability, and reproducibility of AI-assisted surgical planning and outcome prediction.
- Evaluating the impact of AI on plastic surgery precision, patient satisfaction, and overall esthetic outcomes.
- Highlighting limitations, methodological challenges, and areas requiring further research to inform safe and effective integration of AI into clinical practice.

By synthesizing existing evidence, this narrative review aims to provide clinicians, researchers, and other healthcare professionals with a comprehensive understanding of the opportunities and challenges associated with AI in esthetic plastic surgery, ultimately guiding future innovation and adoption in this evolving field.

## 2. Literature Search

A narrative literature search was conducted to identify peer-reviewed studies describing applications of artificial intelligence (AI) in plastic, reconstructive, and esthetic surgery. The search was performed in PubMed/MEDLINE, Embase, and Web of Science from database inception up to 31 December 2025. Searches were limited to English language publications.

Search terms were combined using Boolean operators and adapted to each database. Core AI terms included: “artificial intelligence”, machine learning, deep learning, neural network, convolutional neural network, computer vision, generative adversarial network/GAN, large language model, and ChatGPT. These were combined with plastic surgery terms, including: plastic surgery, reconstructive surgery, esthetic surgery, rhinoplasty, blepharoplasty, facelift/rhytidectomy, breast reconstruction, mammoplasty, facial analysis, 3D imaging, surgical planning, simulation, and outcome prediction.

An example PubMed search strategy is: (“artificial intelligence” OR “machine learning” OR “deep learning” OR “neural network” OR “convolutional neural network” OR “computer vision” OR “generative adversarial network” OR GAN OR “large language model” OR ChatGPT) AND (“plastic surgery” OR “reconstructive surgery” OR “esthetic surgery” OR rhinoplasty OR blepharoplasty OR facelift OR rhytidectomy OR “breast reconstruction” OR mammoplasty OR “facial analysis” OR “3D imaging” OR “surgical planning” OR simulation OR “outcome prediction”).

Titles and abstracts were screened for relevance to clinical or clinically oriented AI use in plastic, reconstructive, or esthetic surgery. Full texts were reviewed when eligibility could not be determined from the abstract. Reference lists of included papers and relevant reviews were hand-searched, and citation tracking was used to identify additional pertinent studies.

**Figure 1:**
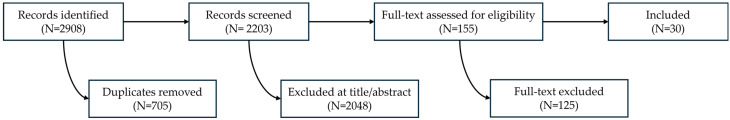
Flow diagram of the literature search and study selection.

Studies were included if they (i) addressed plastic/reconstructive/esthetic surgery contexts and (ii) described AI-based methods for analysis, planning, simulation, prediction, intraoperative support, or patient communication/education. Exclusion criteria included non-surgical cosmetic topics without clear plastic surgery relevance, non-English articles, and conference abstracts lacking sufficient methodological detail. Given the narrative design, no formal risk-of-bias tool was applied; however, emphasis was placed on studies with clear methodology, clinically relevant endpoints, and adequate reporting of datasets and validation. In addition to describing the search strategy, we present a simplified flow diagram summarizing the number of records identified, screened, assessed in full text, and ultimately included in the narrative synthesis (Figure 1).

The topic of our narrative review is inherently conceptual and interdisciplinary, encompassing multiple AI technologies, esthetic philosophies, clinical applications, and surgical subspecialties rather than a single intervention or outcome. The available literature spans heterogeneous study designs, including technical and algorithmic reports, observational clinical studies, proof-of-concept trials, and translational research, with outcomes that are variably defined and often subjective (e.g., esthetic judgment, symmetry, patient satisfaction, surgeon perception). These features preclude formulation of a single, well-defined PICO question and render systematic evidence aggregation methodologically inappropriate. Imposing a systematic review framework would risk artificial homogeneity and misleading precision, whereas a narrative review allows critical appraisal, contextual interpretation, and conceptual integration of technological, clinical, and esthetic dimensions necessary to address the objectives of this article.

## 3. Results

### 3.1. Facelift (Rhytidectomy)

A total of 7 studies about AI and facelift were found and are presented in Table 1.

A study performed by Elliott et al. used a convolutional neural network (CNN) to objectively measure perceived age reduction after facelift surgery in 226 patients, with 96% age estimation accuracy [5]. The AI confirmed significant age reduction sustained up to one year: 3.5 years (5%) at three months and 1.7 years (3%) at twelve months postoperative. Both deep plane and SMAS-plication techniques were effective short-term, but only deep planes showed sustained benefit. Ancillary procedures like fat grafting had strong early effects; benefits diminished over time. No single approach outperformed others long-term [5].

A scientific project coordinated by Du et al. objectively evaluated apparent age (AA) reduction after face-lift surgery in 48 Chinese women using both AI (MiVOLO) and blinded observers [6]. AI demonstrated higher precision (MAE 3.34 years vs. 4.82 years) and stronger correlation with chronological age. The mean AA reduction at one year was significant by both AI (3.75 years) and observers (4.51 years), though patient self-appraisal reported greater reduction. AA improvements reflected preoperative aging status: patients initially appearing older became “back to normal,” while those initially younger looked even younger postoperatively. AI provided an evidence-based, efficient outcome assessment [6].

In their study, Boonipat et al. used AI facial expression analysis on 52 brow lift patients to quantify emotional changes before and after surgery [7]. Post-operatively, the AI detected a significant increase in “happy” emotion (from 1.68% to 9.35%) and significant decreases in “angry” and “sad” emotions (both reduced from 13.06% to ~5.4%). No significant changes occurred in “scared” or “surprise” emotions [7]. Facial action unit analysis showed a decrease in brow lower activation and an increase in upper lid raiser activation, aligning with reduced baseline anger and sadness without provoking a “startled” look. These findings support the use of AI for objective outcome assessment in facial esthetic surgery [7].

Ali et al. evaluated the F4CE app, an AI-driven tool for facial symmetry and proportion analysis using a dataset of 12 female patients [8]. The app accurately detected key facial landmarks and completed 20 measurement tasks per patient, aligning closely with established esthetic standards like the golden ratio. It provided rapid, precise measurements without requiring complex coding skills [8]. Top measurements with minimal deviation included mouth width vs. nose width and face width versus jaw width. Despite the small, homogeneous sample, the study supports F4CE as an efficient, objective tool for pre-surgical planning and postoperative assessment in esthetic practice [8].

A study performed by Gunes et al. suggests high inter-rater agreement with the studied sample of facial beauty perception through a survey of 48 diverse referees rating 215 female faces, demonstrating high agreement [9]. They developed an automated classifier using supervised learning (C4.5) based on facial proportions derived from the golden ratio and facial thirds theories [9]. This classifier achieved high accuracy with error rates below human grading variance, with vertical proportions from the golden ratio being the most predictive [9]. The tool offers an objective method to guide plastic surgery by predicting achievable esthetic outcomes and discouraging excessive interventions [9].

Li et al. developed CLPNet, a deep convolutional neural network based on hourglass architecture, to automatically locate surgical markers for cleft lip and palate repair from 2D facial images [10]. Using a specialized dataset of 2568 labeled images, CLPNet outperformed traditional methods and VGG networks, achieving the lowest mean distance error (MDE 6.91) and failure rate (19.8%). A lightweight version balanced accuracy and real-time efficiency (173 FPS) with significantly reduced model size (13.7 MB) [10]. This AI tool shows promise for improving surgical planning and accessibility, especially in resource-limited settings, though limited by single-center data and labeling subjectivity [10].

A study performed by Gibstein et al. used four CNN neural networks to measure age reduction and patient satisfaction after 105 facelifts using standardized pre- and 1-year post-op photos [11]. AI accurately estimated pre-op age (mean score 100.4). SMAS-ectomy and SMAS-plication showed greater age reductions (5.85 and 5.35 years) than skin-only lifts (2.95 years). Malar fat grafting added 2.1 years to age reduction and significantly improved patient satisfaction per FACE-Q scores [11]. This study confirms AI as a reliable tool for facelift outcome assessment, highlighting better rejuvenation and satisfaction with more invasive techniques and fat grafting [11].

### 3.2. Rhinoplasty

A total of 8 studies describing AI and rhinoplasty were found and are presented in Table 2.

A study performed by Borsting et al. developed RhinoNet, a deep convolutional neural network based on MobileNetV2, to classify rhinoplasty status from 22,686 patient photographs. Tested on 2269 unseen images, RhinoNet achieved 85% accuracy with a sensitivity of 0.84 and specificity of 0.83, statistically equivalent to expert clinicians’ performance [12]. The model’s positive diagnostic likelihood ratio (4.8) was comparable to experts (6.1). Combining AI and human predictions showed potential to enhance diagnostic accuracy [12]. This study highlights the effectiveness of deep learning for visual surgical assessment and the feasibility of mobile AI applications in plastic surgery [12].

Generative Adversarial Network (GAN)-based AI model was developed by a team led by Knoedler et al. and was trained on 3030 paired pre- and postoperative rhinoplasty images, to simulate realistic postoperative outcomes rapidly (within 56 ms) [13]. A survey of 101 participants showed an inability to reliably distinguish GAN-generated images from real postoperative photos, with an accuracy near chance level (52.5%). Male participants identified AI images slightly better than females (55.4% vs. 49.6%) [13]. This GAN model offers a cost-effective, rapid, and highly realistic simulation tool to enhance preoperative patient communication and expectation management, supporting clinical adoption despite current limitations tied to training on a multi-surgeon database [13].

A project undertaken by Chinski et al. developed a GAN-based Artificial Intelligence Model (AIM) trained on 1200 pairs of patient photos and surgeon simulations to generate rhinoplasty outcome images [14]. Excluding complex cases, the AIM generates simulated results within milliseconds from side profile photos. A survey of 97 otolaryngologists showed 68.4% agreement with AIM simulations versus 77.3% agreement with surgeon simulations; this difference was statistically significant [14]. The AIM effectively emulates a surgeon’s esthetic criteria, offering realistic pre-consultation visualizations to aid patient decision-making. Limitations include reliance on a single surgeon’s data, inability to incorporate direct patient feedback, and exclusion of complex surgeries [14].

Dorfman et al. employed a ranking CNN algorithm to objectively quantify the anti-aging effects of open rhinoplasty. Analyzing 100 female patients with postoperative photos at least 12 weeks after surgery, the AI estimated age with high accuracy, showing a strong correlation (r = 0.91) between actual and predicted age. Post-rhinoplasty, patients appeared on average 3.1 years younger compared to preoperative photos [15]. This study is the first to objectively demonstrate the anti-aging effect of rhinoplasty using advanced AI, suggesting its potential integration alongside other facial rejuvenation assessments to better quantify esthetic outcomes [15].

A study performed by Ho Nguyen Anh Tuan et al. developed a hybrid AI model combining a CNN and a back-propagation neural network (BPNN) to predict nasal bone morphology and measurements from 2D photos [16]. The CNN localized 29 facial keypoints with 97.87% accuracy and low error [16]. The BPNN used keypoints, age, gender, and BMI to classify nasal bones in Vietnamese subjects as mostly “V” shaped laterally (78.8%) and Type A frontally (57.6%). BMI correlated with dorsal length and nose width. This non-invasive, radiation-free model aids personalized preoperative nasal analysis and surgical planning [16].

A project undertaken by Suh et al. introduced an AI-integrated augmentation rhinoplasty for Asian patients using virtual 3D surgery software and 3D printing to create customized nasal implants [17]. AI algorithms segment facial structures from 3D CT images and predict nasal cartilage shape, addressing CT limitations [17]. Customized silicone implants match patient anatomy through precise virtual simulation [17]. Clinical results show under 1 mm surgical error, reducing implant deviation and managing complex anatomy like acute radix. This AI-driven approach enhances surgical accuracy, patient satisfaction, and may lower reoperation rates, marking a significant advancement in personalized rhinoplasty planning and execution [17].

A study performed by Štěpánek et al. [18] applied machine learning with geometric facial analysis to evaluate changes in attractiveness and emotion classification relevant to rhinoplasty [18]. Analyzing 42 patient images pre- and post-rhinoplasty, they found a significant mean attractiveness increase of 3.8 Likert points associated with enlargements in the nasofrontal and nasolabial angles [18]. Facial emotions were classified into 14 categories using neural networks, decision trees, and Bayesian classifiers, with neural networks demonstrating the highest accuracy. The geometry of the mouth, eyebrows, and eyes were key features in emotion classification [18].

A research project by Xie et al. evaluated ChatGPT’s capability to simulate an initial rhinoplasty consultation using nine standardized questions [19]. ChatGPT delivered coherent, accessible, and sufficiently informed responses, emphasizing individualized care and advising thorough surgeon consultation [19]. It provided overviews of candidacy criteria, procedural approaches, risks, recovery, and the importance of realistic expectations [19].

### 3.3. Blepharoplasty and Eyelid Surgery

A total of 4 studies regarding AI and blepharoplasty and eyelid surgery were found and are presented in Table 3.

Watane et al. compared ChatGPT-4 and expert Oculofacial Plastic Surgeons (OPS) in answering six common patient questions about upper eyelid blepharoplasty [20]. ChatGPT matched or exceeded OPS performance, scoring higher in comprehensiveness (3.6 vs. 3.0) and showing non-inferior accuracy (3.8 vs. 3.6) and answer similarity (3.2 vs. 2.9). ChatGPT excelled in questions on recovery time and anesthesia [20]. The study supports ChatGPT as a valuable adjunct for patient education and workflow support, especially in low-access settings, while emphasizing it should not replace medical professionals due to limitations like potentially outdated data [20].

A project undertaken by Goodyear et al. employed a deep learning ResNet34 model trained on 47,394 facial images to objectively quantify blepharoplasty’s rejuvenating effects. The model achieved 75% accuracy with a 1.38-year mean absolute error and a high correlation (r = 0.92) [21]. In 103 patients undergoing upper, lower, or quadrilateral blepharoplasty, the model predicted an average age reduction from 0.74 years younger preoperatively to 2.52 years younger postoperatively, indicating about a 2-year rejuvenation effect [21]. Subgroups showed greater effects, such as men undergoing quadrilateral blepharoplasty appearing 5.34 years younger post-op. The study highlights AI’s feasibility in objectively measuring cosmetic surgery outcomes, providing quantitative evidence supporting blepharoplasty’s anti-aging effects [21].

Yixin Qu et al. developed a multichannel CNN model for lower eyelid blepharoplasty, achieving 98.78% accuracy in 3D eyelid reconstruction, surpassing traditional CNN (78.65%) without extra time [22]. In a study of 64 patients (32 AI-assisted, 32 control), the AI group showed significant improvements in pouch degree, skin wrinkles, lacrimal sulcus depth, skin gloss, and esthetic scores, with fewer complications (13% vs. 28%) [22]. This model enhances surgical planning accuracy and esthetic outcomes while reducing complications in eyelid surgery [22].

A research project by Greene et al. compared the clinician-based electronic facial function scale (eFACE) with the machine-learning algorithm Emotrics for assessing eyelid function improvements following eyelid weight placement in facial palsy patients [23]. Their retrospective study of 53 patients showed significant postoperative improvements in eyelid closure and palpebral fissure measures using both tools [23]. eFACE detected synkinesis development in recovering patients, while both assessments demonstrated agreement in functional improvements [23].

### 3.4. Breast Surgery

A total of 11 studies about AI and breast surgery were found and are presented in Table 4.

Kenig et al. demonstrated significant biases in AI-generated esthetic representations using the text-to-image model Craiyon [24]. When prompted with terms like “beautiful breasts,” the AI generated oversized, sexually suggestive images—83% of breast images were oversized and all were deemed sexually suggestive by expert observers with perfect agreement (Cohen kappa = 1) [24]. In contrast, no such biases were found in AI-generated images for males or other body parts [24].

A project undertaken by Chartier et al. introduced BreastGAN, a portable AI tool trained on 1235 paired clinical images to simulate bilateral breast augmentation outcomes [25]. Using a GAN based on the pix2pix framework, BreastGAN generated postoperative images comparable to real surgical results from the senior surgeon’s database [25]. Unlike complex and costly 3D imaging systems, BreastGAN offers a cost-effective, easily deployable alternative, potentially as a mobile app. Model performance improved progressively during training (up to 250 epochs) and GANs can effectively accomplish image translation tasks [25].

A research project by Shiraishi et al. compared ChatGPT-4 (GPT-4) and Grok chatbots in answering clinical questions based on Japanese guidelines for implant-based breast reconstruction [26]. Evaluated by plastic surgery fellows and specialists, GPT-4 significantly outperformed Grok in accuracy, informativeness, and readability. GPT-4’s responses were comparable to the original guidelines per fellow but rated less accurate by experienced specialists, revealing a gap in clinical insight [26]. Grok scored significantly lower than both GPT-4 and guidelines. The study underscores GPT-4’s potential as a clinical aid while cautioning about risks of misinformation and AI limitations, emphasizing the need for specialist oversight and further research [26].

A project undertaken by Bistoni et al. assessed 72 cases of subfascial augmentation mastopexy with P4HB scaffold and smooth round implants, with minimum 12-month follow-up [27]. AI software measured breast parameters, showing minimal inferior pole stretch: 8.04% at 1 year and 9.44% at 3 years [27]. The largest implant volume (>400 cc) correlated with greater stretch (22.17%), while smaller implants (<250 cc) had less (3.81%) [27]. No capsular contracture occurred, and stability was maintained beyond scaffold absorption at 12–18 months, likely due to connective tissue ingrowth. The procedure demonstrated effective long-term support and minimal ptosis recurrence, even with larger implants, confirming the scaffold’s role in stability [27].

A study by Mao et al. used transcriptomic and machine learning analyses to identify key genes and biological mechanisms involved in breast capsular contracture (CC), a common complication after silicone breast implants. Breast capsule tissues from 12 patients were grouped by CC severity (low vs. high) [28]. Machine learning algorithms identified three candidate genes (FGF7, FAM135B, PRKAR2B), with PRKAR2B validated as a novel diagnostic biomarker showing lower expression in severe CC cases and a diagnostic AUC of 0.78 [28].

A research project by Montemurro et al. applied machine learning using Classification and Regression Tree analysis to identify known and novel risk factors for complications in 1625 primary esthetic breast augmentation patients over 11 years [29]. Key findings included the significant role of preoperative bra-cup size larger than A in increasing overall complication risk (odds ratio 2.7) and capsular contracture risk (odds ratio 3.9) [29]. Other important factors were patient height, age, and BMI, with higher BMI strongly linked to capsular contracture, hematoma, and other complications. Round implant shape was also associated with certain complications [29]. The study highlights preoperative breast size as a critical yet previously underappreciated factor in complication risk, supporting its incorporation into patient counseling and surgical planning [29].

A research project by O’Neill et al. developed a machine learning (ML) model using decision tree classification and resampling techniques to predict flap failure in 1012 patients undergoing deep inferior epigastric artery perforator (DIEP) flap breast reconstruction [30]. Flap failure occurred in 1.1% of cases [30]. The model achieved high training accuracy (AUC 0.95) but lower testing accuracy (AUC 0.67), with good specificity (86.8%) but moderate sensitivity (50%). Four high-risk groups were identified with a 7.8% failure rate versus 0.44% in low-risk groups [30]. Key risk factors included elevated BMI (≥30), obesity, comorbidities, smoking, and age ≥ 40. Radiation history was not predictive [30]. This ML approach outperformed conventional regression, highlighting multifactorial causes of flap failure and aiding preoperative risk stratification, although clinical use requires improved sensitivity [30].

A study by Yun et al. evaluated ChatGPT (GPT-4) responses to breast augmentation consultation questions using validated tools and compared scores from plastic surgeons (PS) and laypersons (LP). ChatGPT’s responses had a mean reading level of 10.8, above NIH recommendations [31]. Plastic surgeons rated responses lower than laypersons, especially in reliability and quality, while surgeons scored actionability higher and laypersons valued emotional support more [31]. Scores varied by question type, with consultation and procedure domains rated lower by surgeons due to expectations for technical detail [31]. The study highlighted current evaluation tools as inadequate for assessing AI chatbot quality and emphasized the need for tailored tools to ensure appropriate AI-guided health consultations [31].

Seth et al. evaluated ChatGPT-4’s capacity to provide accurate, comprehensive, and accessible patient information on breast augmentation based on six common questions [32]. The AI delivered well-structured and informative responses aligned with clinical guidelines, covering implant selection, longevity, complications, and costs [32]. While ChatGPT encouraged patients to consult specialists, the model’s generality may limit individualized decision-making [32].

A research project by Grippaudo et al. evaluated ChatGPT’s quality of medical information on common breast plastic surgery procedures (reconstruction, reduction, and augmentation) using the EQIP scale [33]. While all three topics scored above the threshold for quality (19–20/36), the AI showed critical deficits in “Identification data,” failing to cite sources or update information dates [33]. Strengths included clear, well-structured language and logical presentation. However, ChatGPT neglected details like quantitative benefits, risks, procedural sequences, and cost or insurance issues [33].

A research project by Atkinson et al. evaluated ChatGPT-4’s performance in providing intraoperative information during the complex DIEP flap procedure. Four experienced plastic surgeons assessed its responses as medically accurate, systematic, and logical, comparable to trainee knowledge [34]. ChatGPT suggested valid alternative surgical options and approaches to complications like flap compromise, aiding critical thinking. Readability metrics indicated content suited for university-level education [34]. Limitations included occasional lack of detail and context relevance. Overall, ChatGPT shows promise as a supplementary clinical decision support tool that can reduce surgeon cognitive load during complex surgeries, though it needs further refinement to address individual patient factors fully [34].

## 4. Discussion

This narrative review summarizes current evidence on artificial intelligence (AI) applications in esthetic plastic surgery and highlights both the clinical opportunities and the limitations that currently restrict broad implementation. Across the included studies, AI tools were most frequently applied to preoperative image analysis, automated landmark detection, quantification of symmetry and proportionality, simulation of potential postoperative appearances, and prediction of outcomes or complication risk. Collectively, these approaches suggest that AI can support more standardized assessment and planning, while providing an additional layer of data-driven decision support alongside surgeon expertise.

A key contribution of AI in esthetic practice is the potential to reduce variability in how outcomes are assessed and communicated. Traditional evaluation relies heavily on subjective judgment and inter-observer interpretation. In contrast, computer vision and deep learning methods can quantify anatomical landmarks and esthetic proportions in a reproducible manner, which may improve comparability between cases and support more consistent counseling. In addition, predictive models trained on clinical and imaging datasets may help estimate postoperative changes or complication risks and identify patient subgroups more likely to require revision procedures, enabling more individualized planning and shared decision-making.

Beyond measurement and prediction, AI-based simulation and generative models may enhance preoperative consultations by allowing patients to visualize potential results and align expectations with anatomically plausible outcomes. When used responsibly, simulation can support informed consent and reduce dissatisfaction driven by unrealistic expectations. However, simulation outputs should be presented as probabilistic and illustrative rather than deterministic, because model performance is dependent on the training data and may not generalize to diverse populations, imaging conditions, or surgical techniques.

Despite encouraging signals, the current evidence base remains heterogeneous and often methodologically limited. Many studies are retrospective, involve small or single-center datasets, and use inconsistent outcome measures and reporting standards, which constrains direct comparison across studies and limits generalizability. External validation is frequently absent, and performance reported in development cohorts may not translate to real-world settings. Furthermore, many AI models function as “black boxes”, raising concerns regarding interpretability, accountability, and how outputs should be integrated into clinical decision-making. These issues are particularly relevant in esthetic surgery, where patient preferences and psychosocial factors influence both indications and satisfaction.

The rapidly expanding literature on artificial intelligence applications in esthetic and plastic surgery remains highly fragmented and methodologically heterogeneous, which has important implications for how results can be synthesized and presented. Across studies, there is substantial variability in study design, sample size, clinical context, and reporting standards, with many investigations emphasizing proof-of-concept development, algorithmic feasibility, or technical performance rather than standardized clinical evaluation. Objective measurement approaches, including facial landmark detection, symmetry analysis, and age estimation—are characterized by non-uniform definitions, proprietary or institution-specific datasets, and bespoke annotation protocols, limiting direct comparability across studies. Predictive models addressing surgical outcomes or complications differ markedly in endpoint selection, follow-up duration, and covariate structure, and frequently lack comprehensive reporting of calibration, discrimination metrics, or statistical significance. Simulation and generative models intended for esthetic outcome visualization or expectation management are similarly heterogeneous, often prioritizing technical capability over validated clinical performance, with limited assessment of failure modes, bias, or patient-centered outcomes. Critically, key methodological elements required for analytical cross-study synthesis—such as consistent outcome measures, standardized validation strategies (internal versus external), and harmonized performance metrics—are not uniformly reported across the literature. As a result, imposing a more formal analytical structure within the Results section would necessitate speculative harmonization or selective interpretation beyond the primary data, introducing a risk of artificial coherence and potentially misleading conclusions. In this context, a narrative synthesis that transparently reports available data as presented in the original studies, while deferring higher-level integration and critique to the Discussion, represents the most methodologically appropriate approach. Consistent with this rationale, summary tables report sample size, validation type, key metrics, and statistical significance only when explicitly provided by the source publications; where such information is absent, this is clearly indicated. This approach preserves fidelity to the underlying evidence base and accurately reflects the current developmental stage of AI-driven esthetic applications, while underscoring the need for more standardized reporting, external validation, and clinically meaningful outcome measures in future research.

Ethical and regulatory considerations are central to implementation. AI systems trained on restricted or non-representative datasets may embed bias, potentially worsening inequities in assessment or predicted outcomes across age groups, sexes, or ethnic backgrounds. Privacy and consent are also critical, given the sensitivity of facial and body images and the risks of secondary data use. Clear governance, transparent reporting, secure data handling, and clinician oversight are essential to prevent misuse, overreliance, or misinterpretation of algorithmic outputs.

Future work should prioritize prospective, multi-center studies with larger and more diverse cohorts, standardized definitions of outcomes, and transparent reporting of datasets, preprocessing, and validation procedures. Incorporating patient-reported outcome measures alongside objective metrics will be important to capture the patient-centered value of AI-assisted planning and assessment. Finally, collaboration between plastic surgeons, data scientists, biostatisticians, and ethicists will be necessary to ensure that tools are clinically meaningful, appropriately validated, and implemented in a way that supports—rather than replaces—clinical judgment.

In summary, AI is a promising adjunct in esthetic plastic surgery, with potential benefits in measurement, planning, simulation, and outcome prediction. However, the current literature supports cautious integration, emphasizing external validation, transparency, bias mitigation, and ethical governance before widespread adoption in routine clinical practice.

## 5. Conclusions

AI is emerging as a powerful tool in esthetic plastic surgery, offering a potential to enhance precision, improve outcome predictability, and support individualized surgical planning. Current evidence demonstrates promising applications in image analysis, surgical simulation, and outcome prediction; however, variability in study design, limited sample sizes, and lack of standardized metrics underscore the need for further high-quality research. Future studies should focus on rigorous validation, integration of patient-reported outcomes, and ethical implementation to fully realize the benefits of AI in clinical practice. By bridging technology and surgical artistry, AI has the potential to redefine the standards of precision and beauty in esthetic plastic surgery.

## Figures and Tables

**Table 1:** Summary of results for rhytidectomy.

Author (Year)	Algorithm	Number of Patients/Images	Main Findings
Elliott et al. (2023) [5]	Convolutional Neural Network (CNN)	226 patients that underwent facelift surgery between 2017 and 2021	- CNN predicted age with 96% accuracy but overestimated by 2.5 years - 3 months post-op: 3.5-year age reduction (*p* ≤ 0.001) - 12 months post-op: 1.7-year reduction (*p* ≈ 0.03) - No prior surgery: 4.1 years younger at 3 months, 2.2 years at 12 months - Prior surgery: 3.1 years younger at 3 months, minimal effect at 12 months - Deep Plane and SMAS-plication: equally effective short-term - Fat grafting: significant benefit at both times (4.3 and 2.5 years) - Blepharoplasty: significant only at 3 months (4.1 years) - Males: 2.3-year reduction at 3 months, not significant
Du et al. (2024) [6]	Artificial neural network	Standardized pre- and postoperative images of 48 female patients	- AI measured 3.75 years younger; observers 4.51 years; patients felt 7.3 years younger - AI more precise with lower error and higher correlation to actual age. - Patients either looked older pre-op than normalized post-op, or looked younger pre-op and got even younger post-op with surgery - No significant difference in AI or observer age reduction between patient groups - Average age 47 years; 6.25% minor complications; 35% had additional procedures (mostly blepharoplasty)
Boonipat et al. (2021) [7]	Artificial neural network	pre and postoperative images of 52 patients going for bilateral browlift surgery	- Appearance of “angry” emotion decreased from 13.06% (pre-op) to 5.42% (post-op) - Appearance of “happy” emotion increased from 1.68% (pre-op) to 9.35% (post-op) - Both statistically significant
Ali and Cui (2025) [8]	Artificial neural network	publicly available dataset of 12 female patients	- F4CE app accurately detected facial landmarks for all 12 patients. - It precisely measured facial symmetry and proportions. - Measurements aligned closely with established esthetic standards, especially the golden ratio. - No statistically significant differences were found.
Gunes and Piccardi (2006) [9]	Supervised symbolic classifier	215 female facial images	Classifiers achieved high accuracy in reproducing the average human judgment, with statistical significance
Li et al. (2019) [10]	Artificial neural network	Surgical Markers for Complete Cleft Lip (SMCCL) database: 2568 facial images	Strong superiority and adaptability of the specialized deep learning methods on all criteria relative to a general deep learning model. Statistically significant.
Gibstein et al. (2021) [11]	Artificial neural network	105 female patients with anterior and lateral images pre and postoperative (1-year): ancillary technique or facelift technique	- SMAS facelift techniques (SMAS-ectomy and SMAS-plication) reduced estimated age by ~5.35–5.85 years, significantly more than skin-only lifts (~2.95 years) (*p* < 0.05). - No significant difference between SMAS-ectomy and SMAS-plication techniques. - Fat grafting added an extra 2.1 years of rejuvenation (5.88 vs. 3.78 years, *p* < 0.05). - Patient satisfaction was higher with fat grafting but similar across facelift types.

**Table 2:** Summary of results for rhinoplasty.

Author (Year)	Algorithm	Number of Patients/Images	Main Findings
Borsting et al. (2020) [12]	Artificial neural network	2269 previously unseen test-set images of rhinoplasty	Correctly predicted rhinoplasty status in 85% of the test-set images. Sensitivity: 0.84 (0.79–0.89). Specificity: 0.83 (0.77–0.88). Statistical significance not stated
Knoedler et al. (2024) [13]	Generative Adversarial Network (GAN)	3030 rhinoplasty patients’ pre- and postoperative images + 101 study participants	- GAN produced highly realistic images; evaluators identified AI-generated photos correctly in 52.5% ± 14.3% of cases - Males performed slightly better (55.4% ± 14.4%) than females (49.6% ± 13.7%), *p* = 0.04. - No significant difference based on surgical experience (*p* = 0.26), prior consideration of surgery (*p* = 0.72), or age (</> 31.6 years; *p* = 0.82).
Chinski et al. (2022) [14]	Generative Adversarial Network (GAN)	1200 pairs of original and surgeon-simulated profile images of patients who consulted for esthetic primary rhinoplasty.	- AIM successfully emulated the surgeon’s esthetic criteria to generate simulated images. - Median agreement score for the AIM simulation was 5 (vs. 6 for the surgeon’s simulation, *p* < 0.0001). - Total or partial agreement (scores 5–7) with the AIM simulation was achieved 68.4% of the time. - The software generates a simulated image in a matter of milliseconds after training. - The tool can provide patients with a realistic approximation of possible results ahead of an in-person consultation.
Dorfman et al. (2020) [15]	Convolutional Neural Network (CNN)	Standardized post-op photos of 100 women who underwent rhinoplasty	- The ranking CNN accurately estimated preoperative age, with a correlation coefficient of r = 0.91. - On average, patients were predicted to look 3 years younger post-open rhinoplasty. - The objective age reduction was statistically significant (*p* < 0.0001). - The CNN algorithm eliminated the subjective error inherent in previous studies relying on human observers. - The anti-aging effects of open rhinoplasty should be revisited and considered alongside other facial rejuvenation procedures.
Ho Nguyen Anh Tuan et al. (2022) [16]	Convolutional Neural Network (CNN) and Back-Propagation Neural Network (BPNN)	2000 digital 2D facial images, 182 living participants, and 33 cadavers	- The CNN model achieved 97.869% accuracy in facial key point localization. - In the Vietnamese population, 78.8% displayed a “V” shaped nasal bone in the lateral view. - Type A nasal morphology was most common (57.6%) in the frontal view. - A back-propagation neural network found BMI correlates with nasal features: dorsal length was longer in underweight/normal weight groups, while nose width was greater in overweight/obese groups.
Suh, Won, and Baek (2024) [17]	Virtual 3D Surgery Software/Deep Learning (Snake, U-net)	3D CT images of 4 rhinoplasty patients	- Postoperative outcomes matched the 3D surgical plan with high accuracy - Patient-specific implants corrected deviations based on anatomical asymmetry - Deep learning algorithms segmented CT data to predict nasal cartilage shape for precise implant design - Customized implants minimized dissatisfaction by aligning with virtual surgery-confirmed patient wishes - The system achieved surgical prediction errors below 1 mm through AI learning
Štěpánek, Kasal, and Měšťák (2020) [18]	Artificial neural network + Bayesian naive classifiers + decision trees (CART)	30 patients who underwent rhinoplasty surgery + 168 pictures, each showing a facial expression	- Attractiveness Prediction: Enlargements of both a nasolabial and nasofrontal angle within rhinoplasty were determined as significant predictors increasing facial attractiveness. Mean increase in attractiveness after rhinoplasty = 3.8 Likert points. Statistically significant. - Neural networks manifested the highest predictive accuracy for classifying a new face into facial emotions, statistically significant
Xie et al. (2023) [19]	Artificial neural network	9 hypothetical questions simulating an initial consultation about rhinoplasty	ChatGPT provided coherent and easily comprehensible answers. The responses were sufficiently informed, recognized their limitations in providing more detailed, personalized, or esoteric advice. Statistical significance not stated

**Table 3:** Summary of results- AI applications in blepharoplasty and eyelid surgery.

Author (Year)	Algorithm	Number of Patients/Images	Main Findings
Watane et al. (2025) [20]	Artificial neural network	6 FAQs about upper eyelid blepharoplasty, 36 answers collected in total from OPS and ChatGPT	- ChatGPT was found to be superior to OPS - Accuracy score without statistically significant difference - Comprehensivness with statistically significant difference in favor of ChatGPT - Personnal answer similarity without statistically significant difference
Goodyear et al. (2023) [21]	Artificial neural network	299 photographs from 103 patients who underwent blepharoplasty	- Overall rejuvenation: Apparent age decreased from 0.74 years younger pre-op to 2.52 years younger post-op → ~2-year rejuvenating effect - Statistical significance: *p* < 0.001 (significant difference between pre- and postoperative predictions) - Women (lower blepharoplasty, n = 23): 1.28 years older pre-op → 2.32 years younger post-op (*p* = 3.8 × 10^−4^) - Men (quadrilateral blepharoplasty, n = 10): 0.71 years younger pre-op → 5.34 years younger post-op (*p* = 0.02)
Yixin Qu et al. (2022) [22]	Multichannel Convolutional Neural Network (CNN)	64 patients underwent pouch plastic surgery.	- MCNN eye shape reconstruction had 98.78% similarity to actual shape, outperforming traditional CNN at 78.65% - Patients assisted by the CNN model had better esthetic outcomes post-surgery - The CNN-assisted group showed greater improvement in surgical indicators (pouch degree, skin wrinkles, skin gloss) than controls. - Complication rates were lower in the CNN group (13%) compared to controls (28%) - Reconstruction time was similar between MCNN (3.41 s) and traditional CNN (4.02 s) with no significant difference
Greene et al. (2019) [23]	Emotrics + clinician tool	53 patients with unilateral facial palsy who received an eyelid weight placement	In Whole cohort subgroups, for both systems all parameters improved with statistical significance post-op In the no-expected recovery subgroup almost no parameter reached statistical significance (small sample)

**Table 4:** Summary of results- AI applications in breast surgery.

Author (Year)	Algorithm	Number of Patients/Images	Main Findings
Kenig et al. (2023) [24]	Artificial neural network	90 images of breasts generated by Craiyon	- 90 breast images reviewed by two expert observers showed 100% sexual suggestiveness with perfect agreement - 83% of “beautiful breasts” images were classified as oversized; no images were undersized - Mixed sexual suggestiveness in male images, more noted by female observer - AI’s bias toward portraying female breasts as oversized and sexualized, likely reflecting societal stereotypes
Chartier et al. (2022) [25]	Artificial neural network	pre- and postoperative images of 1235 patients who have undergone bilateral breast augmentation	- Offers a cost-effective alternative to bulky, expensive 3D imaging - Model performance improved progressively during training (up to 250 epochs) - GANs can effectively accomplish image translation tasks - Empower patients by helping visualize potential surgical results, facilitating realistic expectation - No significance values stated
Shiraishi et al. (2025) [26]	Artificial neural network	5 clinical questions from the Japanese Practical Guidelines for Implant-based Breast Reconstruction (IBBR)	- GPT-4 outperformed Grok with statistical significance - Grok consistently was inferior to guidelines with statistical significance - There is a statistically significant difference in how fellows and specialists marked the accuracy of the AI, fellows marking it significantly higher
Bistoni, Sofo, Cagli, Buccheri, and Mallucci (2024) [27]	3D Artificial intelligence software	72 patients who underwent subfascial augmentation mastopexy with P4HB scaffold.	- No recurrent ptosis, bottoming out, implant displacement, or capsular contracture reported over a mean 24.8-month follow-up. - Lower Pole Arch (LPA) elongation was significantly greater at 6 and 12 months than at 6 weeks (*p* < 0.0001). - Sternal notch to nipple distance (SN-N) remained stable at 12 months (*p* = 0.242). - Larger implants (>400 cc) caused significantly more LPA stretch than smaller ones (200–250 cc) (*p* = 0.0011). - LPA elongation was minimal (8.04% at 1 year, 9.44% at 3 years), supporting the P4HB scaffold’s role in long-term lower pole stability.
Mao et al. (2023) [28]	Support Vector Machine Recursive Feature Elimination (SVM-RFE) and Least Absolute Shrinkage and Selection Operator (LASSO)	RNA sequencing from 15 breast capsule samples from 12 female patients, categorized by Baker grade (LCC/HCC)	- Machine learning identified PRKAR2B as a novel diagnostic biomarker. - PRKAR2B expression was lower in high capsular contracture (HCC) groups (*p* < 0.01). - The diagnostic effectiveness of PRKAR2B yielded an Area Under the Curve (AUC) of 0.786. - HCC exhibited significantly higher proportions of M1 macrophages (*p* < 0.05) and follicular helper T cells (*p* < 0.01). - The cGMP–PKG and PI3K–Akt signaling pathways were implicated in CC fibrosis progression
Montemurro, Lehnhardt, Behr, and Wallner (2022) [29]	Classification and Regression Tree (CART) analysis.	1625 female patients who underwent primary esthetic breast augmentation by a single surgeon between 1/2010 and 12/2021	- Preoperative bra-cup size larger than A was the most crucial factor for overall complications (OR 2.7, *p* < 0.0001). - Capsular contracture risk was highest with higher BMI, larger than A preoperative bra-cup size (OR 3.9, *p* = 0.0004), and higher age. - Taller body height was identified as a significant factor influencing overall complications and implant rupture. - The decision tree model showed high accuracy (e.g., Capsular Contracture Testing Accuracy 98.2%). - The study confirmed known risks (high BMI, round implants) and identified previously unconsidered factors (preoperative breast size).
O’Neill et al. (2020) [30]	Decision tree model and Random Oversampling Examples (ROSE) technique.	1012 patients who underwent microvascular breast reconstruction using deep inferior epigastric artery perforator (DIEP) flaps	- Total flap failure occurred in 1.1% of patients (12 cases). - The ROSE/decision tree model achieved a strong prediction (AUC 0.95 in training; 0.67 in testing). - The model identified four high-risk patient subgroups, whose failure rate was 7.8% (compared to 0.44% in low-risk patients). - Increased BMI (obesity), comorbidities, and smoking were found to contribute synergistically to flap loss. - The algorithm suggested flap failure is multifactorial, recognizing patterns that traditional logistic regression failed to identify.
Yun, Kim, Lee, and Kim (2023) [31]	Artificial neural network	25 questions/responses by ChatGPT simulating mammoplasty consultations	Plastic Surgeons (PS) vs. Laypersons (LP): - PS scored lower than LP on all DISCERN domains (reliability, quality, overall, *p* ≤ 0.014) - PS rated emotional tone lower than LP (*p* = 0.002) - PS scored higher than LP on PEMAT actionability (*p* = 0.013) - No difference in PEMAT understandability (*p* = 0.051)
Seth et al. (2023) [32]	Artificial neural network	6 commonly asked questions regarding breast augmentation	Responses were relevant and accurate in most cases, lacked personalization and sometimes generated inappropriate or outdated references. Statistical significance not stated
Grippaudo et al. (2024) [33]	Artificial neural network	Questions regarding 3 common procedures in breast plastic surgery	Total Mean Scores (out of 36) evaluated via expanded EQIP scale: Breast reconstruction: 19/36. Breast reduction: 19/36. Augmentation Mammaplasty: 20/36. Statistical significance not stated
Atkinson et al. (2024) [34]	Artificial neural network	A series of six intraoperative questions specific to the deep inferior epigastric artery perforator (DIEP) flap procedure	Responses were found to be medically accurate, systematic in presentation, and logical when providing alternative solutions. The responses corresponded to the knowledge level of a plastic surgery trainee. No statistical significance stated

## Data Availability

No new data were created or analyzed in this study.

## Abbreviations

The following abbreviations are used in this manuscript:

2D	two-dimensional
3D	three-dimensional
AA	apparent age
AI	artificial intelligence
AIM	artificial intelligence model
AUC	area under the curve
BMI	body mass index
BPNN	back-propagation neural network
CC	capsular contracture
CNN	convolutional neural network
CT	computed tomography
DIEP	deep inferior epigastric perforator (flap)
EQIP	Ensuring Quality Information for Patients (EQIP) tool
FACE-Q	Facial Clinimetric Evaluation questionnaire
FPS	frames per second
GAN	generative adversarial network
GPT-4	Generative Pre-trained Transformer 4
LP	laypersons
MAE	mean absolute error
MDE	mean distance error
ML	machine learning
NIH	National Institutes of Health
OPS	oculofacial plastic surgeons
PS	plastic surgeons
SMAS	superficial musculoaponeurotic system
VGG	Visual Geometry Group (CNN architecture)
